# The individual and ecological characteristics of parental COVID-19 vaccination decisions

**DOI:** 10.1038/s41598-024-74963-8

**Published:** 2024-10-15

**Authors:** Lukas Hörnig, Sandra Schaffner, Hendrik Schmitz

**Affiliations:** 1 RWI – Leibniz-Institute for Economic Research, Essen, Germany; 2 Ruhr-University Bochum, Bochum, Germany; 3 Paderborn University, Paderborn, Germany; 4 Leibniz Science Campus Ruhr, Essen, Germany

**Keywords:** COVID-19 vaccination, Vaccine hesitancy, Parents, Children, Adolescents, Epidemiology, Epidemiology, Risk factors, Viral infection

## Abstract

Vaccination is a highly effective method to prevent the spread of COVID-19 and mitigate severe disease. In Germany, adult vaccination rates are relatively high at 85.5%, but rates are significantly lower for adolescents (69.6%) and children (20.0%). This discrepancy indicates that not all vaccinated parents choose to vaccinate their children. Analyzing data from a January 2022 online survey of 1,819 parents with children and adolescents, we explore the socio-economic factors influencing parents’ willingness to vaccinate themselves and their children. Our results show that individuals who vote for either side of the political extremes are less likely to vaccinate their children. This pattern is particularly strong for voters on the far right. In addition, we find that better educated parents are more likely to vaccinate both themselves and their children. Parents who vaccinate both themselves and their children demonstrate greater confidence in the vaccine’s effectiveness, while those who vaccinate only themselves are often motivated by a desire to regain personal freedoms. These insights highlight the need for targeted public health strategies to address specific concerns and improve vaccination rates among children and adolescents.

**Supplementary Information:**

The online version contains supplementary material available at 10.1038/s41598-024-74963-8.

## Introduction

Children have been assumed to be important drivers of COVID-19, particularly at the beginning of the pandemic^1,2^. While this view was challenged later by many others^3^, it most likely has contributed to school closures as an important policy measure in 2020 and 2021 in almost all countries worldwide. Irrespective of whether school closures have been effective measures – as there are conflicting studies finding a relationship^4–6^and studies not finding a relationship in Germany^7,8^– policymakers and scholars seem to have converged on the opinion that school closures should be prevented in the future^9^. On the other hand, open schools (and compulsory schooling) have raised concerns about safe learning environments during a pandemic, particularly in families with high-risk individuals.

The potentially most fruitful way out of this dilemma is vaccination, which either decreases the number of infections and/or, more importantly, together with the Omicron variant of the Corona virus reduces the burden of disease and mortality. Vaccination against COVID-19 in Germany started in December 2020 for adults and was expanded to children between the ages of 12 and 17 years in August 2021. Children between 5 and 11 years of age have been vaccinated since November 2021. However, while vaccination rates are comparably high for adults at around 83% (18–59 years), they are substantially smaller for children: 70% of children aged 12–17 years old and only 20% of children aged 5–11 years old children had basic immunization in 2023^10^.

If these numbers are to be increased, the characteristics of parental willingness to vaccinate their children need to be better understood. The aim of our study is to contribute here. A considerable amount of research has been conducted to describe child vaccination against COVID-19, which, however, is much less than that regarding adult vaccination. While quantitatively most of the studies on parental willingness to vaccinate their children seem to be from the Asian continent, there are also several European studies for Denmark^11^, England^12^, Germany^13^, Denmark, Germany^14^, Italy^15–18^, and Poland^19^. Goldman et al. (2020) conduct a study in several countries^20^. Furthermore, there are some studies from the U.S^21,22^.

Relevant factors associated with child vaccination seem to be the age of the parents, with older parents being more likely to vaccinate their children^13,16,20,23–27^. Moreover, men seem to have a higher willingness to vaccinate their children than women^16,20,23,24,26,28–30^, although there is opposing evidence^19^. Higher income and educational level are also associated with a higher willingness to vaccinate their children^31–36^. However, other studies have found the opposite^6,24,27,37–39^.

The age of the child^14,16,17,20^and the fact that parents work in the health sector^15,19,25,27,39^also increases vaccination readiness. In addition, ethnic background appears to be a factor, as some authors have found that Black, Asian, and other ethnic minorities are more likely to refuse vaccination^12,25,27^.

Only a few studies directly relate parental vaccination to child vaccination. Unsurprisingly, own (parental) vaccination status is shown to be a major determinant of child vaccination^40,41^, probably due to a generally positive attitude towards vaccination. However, the vaccination rates cited above leave the impression that there must be many parents who vaccinate themselves, but not their children. This paper aims to describe this group and the reasons for (not) vaccinating their children. Our specific research questions were as follows.

- What are the socioeconomic and ecological characteristics of parents who are willing to vaccinate themselves and their children against COVID-19? Does the experience of restrictions due to Corona measures matter for vaccination decisions?
- Who are the parents that vaccinate themselves but not their children?
- What are the stated reasons of vaccinated parents not to vaccinate their children?
- What are the socioeconomic characteristics of parents who state not to vaccinate their children due to fear of side effects or a general lack of trust^1^?

Understanding the correlates of vaccination decisions is the basis for addressing the problem of too low vaccination rates in children between 5 and 11. An important socioeconomic characteristic we study is education. We hypothesize that lower educational status is associated with a lower willingness to vaccinate in general. However, when restricting the group to vaccinated parents, it is not clear whether education is also correlated with the decision not to vaccinate their children.

## Data and methods

We use data from the third wave of the Casa Monitor Smaragd Survey. The Smaragd Survey is an online survey conducted by infas360^42^ with an online-representative sample of 10,000 participants in Germany. The data are representative of the federal state. We received data from infas360 on March 17th, 2022. infas360 assigns randomized identifiers, making it impossible to identify individual participants. The survey was conducted between January 3rd and January 31st, 2022, and respondents voluntarily participated. Infas360 has a pool of pre-registered target persons (Casa Monitor) who representatively reflect the German online population aged 18 years and over. Infas360 assigned participants to the survey until 10,000 individuals completed it (opt-in non-probability sampling). To ensure representativeness, infas360 uses blended calibration, which integrates non-probability samples with probability-based surveys to reduce sampling error in population estimates. Despite these efforts, some sampling error may remain, potentially biasing our subsequent inference.

To become part of the online panel, potential participants must give written consent to the data privacy policy (see https://mingle.respondi.de/privacy-policy. The participants are informed about the use for scientific research, anonymization is through this data privacy policy. Since participants get individual links they cannot participate several times. The participants were informed that the survey did not trigger powerful emotions or physical pain. Hence, the Economic Ethics Committee of the University Alliance Ruhr and RWI approved our research (certificate No. 2024_1_SS). We confirm that all experiments were performed in accordance with relevant guidelines and regulations. Participants were not informed of the topic of the survey prior to participation. Exact response rates are not available for our specific survey, but the Casa Monitor typically has a response rate between 30 and 40%. Participation is incentivized with €0.25 per 5 min. Participants in the third wave received an average of €0.50, so the average duration was about 10 min.

The questionnaire of the survey can be found in the FDZ Data Description^43^. It was possible to choose not to answer any question and continue with the questionnaire. As (child) vaccinations were a controversial topic at the time, there could be a selection bias in terms of who participated in the survey. In the Appendix, we compare the descriptive statistics of the respondents to administrative information and show that they are very similar in terms of demographics and regional coverage (see Table A1). Hence, we do not observe a strong selection, at least in terms of observables. The survey sampled individuals but not households. This means that we learn about the vaccination intentions of one parent but not of the other. Yet, the decision to vaccinate children is likely to be made jointly by both parents. While actual vaccination status is asked for adolescents, we believe that this is not an issue here. Yet, vaccination willingness is asked for children under 12. The own opinion of one parent, then, does not necessarily reflect the eventual vaccination behavior of the household.

### Sample selection

A total of 10,000 individuals aged between 18 and 90 years participated in the survey. We restrict the sample to parents who are, at most, 60 years old and have children younger than 18 years. While we observe whether respondents have children, we do not know the exact age of the children (only whether they are below 12 or 12–17). We exclude individuals who reported not to vaccinate their children because they were too young, thereby assuming that their children were below five years old. However, it is unclear whether some parents may not have sufficient knowledge of vaccination regulations, despite media coverage being prevalent on the topic. Parents with children under age 12, who report their children as too young, have a median age of 34 years, compared with 38 years for those who do not. This suggests that the parents were well informed. All in all, 1,819 parents are included in the final sample. 1,051 of them have children below 12 years of age, whereas 1,145 had children aged 12–17 (we do not exclude parents who have children in both age groups).

Unfortunately, our sample does not include information on how many children the respondents have within the two categories 5–11 and 12–17. Possibly, parents have two children aged 5–11 and have one child vaccinated but not the other. According to our auxiliary analysis with data from the German Microcensus of the year 2020 that covers around 400.000 individuals, among all households with children between 5 and 17, 70.7% have either exactly one child between 5 and 11 (29.4%), one child between 12 and 17 (29.2%) or one child 5–11 and one child 12–17 (12.1%). The remaining 29.3% have at least two children in one of the two age groups.

### Outcome

The survey questions on vaccination (directed to the parents) read as follows:

- Have you been vaccinated against the Corona virus? Possible answers: Yes or No.
- Have you vaccinated your children (between 12 and 17)? Possible answers: Yes or No.
- Would you like to have your children under 12 vaccinated? Possible answers: Yes (as soon as possible), not yet decided, no.

While vaccination for adults and 12–17 year was already possible in January 2022, it was not yet universally available for all children below 12. While vaccinations were approved for this age group in Germany in November 2021, not every child had received an actual offer at a local vaccination center in January 2022. Thus, the third question is hypothetical, while the first two questions are about actual vaccination status.

**Fig. 1:**
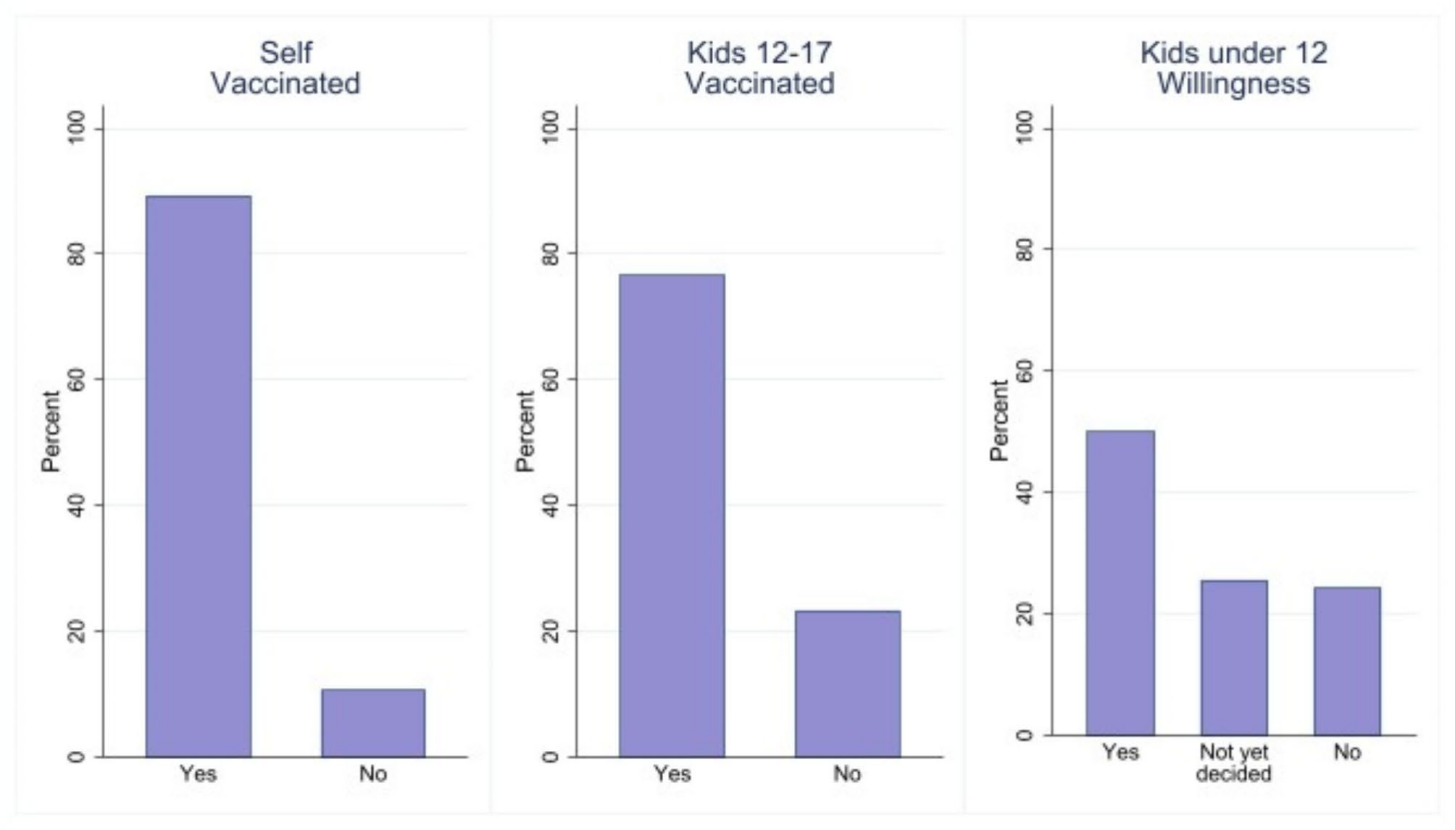
Distribution of vaccination willingness. Smaragd Survey, Wave 3, January 2022. The left panel only includes parents with children below 18 (*n* = 1,819). The middle panel only includes parents with children between 12 and 17 (*n* = 1,145). The right panel only includes parents with children under 12 (*n* = 1,051).

The responses are shown in Fig. 1. Approximately 90% of parents in the sample are vaccinated at least once. This finding is consistent with the average vaccination rate in Germany. Moreover, 75% of children aged 12–17 are vaccinated. This is in contrast to the stated willingness to vaccinate children between 5 and 11 years of age, once a vaccine is available (which is the case as of today but has not been so for everybody in January 2022). In our sample, 50% are planning to vaccinate their children, while one-fourth has not decided yet and another fourth plans not to vaccinate. Interestingly, a willingness of 50% is still significantly higher than the official vaccination prevalence, which was around 20% in July 2024. We use this stated willingness as an important dependent variable, keeping in mind that this differs from actual vaccination behavior. As long as the difference between stated and actual preferences does not systematically vary by socioeconomic status, we do not consider this a fundamental problem^44^.

### Institutional setting

On January 27, 2020, Germany reported its first case of the new coronavirus. From March, the German government implemented measures to control the spread of the virus, including recommending the cancellation of events with more than 1,000 participants and imposing travel restrictions. On March 13, all state governments decided to close schools from March 16 to April 19, 2020. A minimum distance of 1.5 m was imposed in public places and people were only allowed to be in public places with one other person from outside their household. Restaurants and many service businesses were closed. Restrictions were gradually lifted under strict hygiene and distance rules, and primary schools in North-Rhine Westphalia (NRW), the largest federal state – to name one example – reopened on 4 May. Pupils returned to normal schooling on a rotating basis before the summer holidays.

As infections increased, a “lockdown light” was introduced in November 2020, with restaurants, bars and gyms closed and private gatherings limited to 10 people. The lockdown was tightened further, and primary schools in NRW were again closed from mid-December to the end of February 2021, along with most shops and service businesses. In March 2021, a gradual easing of the lockdown began, depending on local infection rates. From 23 April to 30 June 2021, the Federal Emergency Brake was in effect, imposing uniform measures based on local incidence rates, including curfews and alternative day schooling for high incidence areas.

Adjustments to testing and infection control measures were agreed on 10 August 2021. Access to many public places required proof of vaccination, recovery or a negative test (the so-called “3G rule”). Free public testing ended on October 11, but was reintroduced on November 13. Large events were allowed with hygiene plans.

On November 19, 2021, new infection control measures were approved, including a home office mandate where possible and a 3G rule in workplaces otherwise. Mandatory testing was introduced for staff and visitors to retirement homes, nursing homes and healthcare facilities. The 3G regulation also applied to local and long-distance transport and domestic flights. States could continue measures such as mandatory masks and contact restrictions, but could no longer impose nationwide exit restrictions, travel bans or school and business closures. Stricter measures were linked to hospitalization rates, with the introduction of 2G (vaccinated or recovered) and 2G-plus (vaccinated or recovered plus testing) rules depending on the severity of hospitalization rates.

Corona mitigation measures increasingly pressured unvaccinated people by requiring regular testing. Vaccination in Germany began on December 27, 2020, but initially vaccines were scarce, and priority was given to the elderly and those in high-risk occupations. On June 7, 2021, this prioritization was lifted, but demand still outstripped vaccine supply. In June, the STIKO (Standing Committee on Vaccination) recommended vaccination for children aged 12–17 only if they had pre-existing conditions (such as adiposity or immunodeficiency) or were in close contact with a high-risk person. The vaccine could be given to children without pre-existing conditions if they wanted it and their parents accepted the risks. Two months later, STIKO recommended the vaccine for all children aged 12 to 17. For younger children, ages 5 to 11, STIKO waited until early January 2022 to recommend the vaccine, again only for those with pre-existing conditions or in close contact with a high-risk person. As before, vaccination was possible without a pre-existing condition if parents accepted the risks.

The vaccination campaign against COVID-19 received broad support across most political parties. Until the end of 2021, the government was formed by the Christian Democrats (CDU/CSU) and the Social Democrats (SPD), who actively promoted vaccinations as a critical tool in combating the pandemic. The opposition parties, the Greens (Grüne) and the Free Democrats (FDP), also supported the vaccination efforts. Their primary criticism was not of the campaign itself but of its pace, arguing that the government needed to accelerate the rollout to reach more people more quickly. After joining the government with the SPD in December 2021, the Greens and FDP maintained their commitment to the vaccination campaign, emphasizing its importance in ensuring public health.

The Left Party (Die Linke) also supported the vaccination campaign, recognizing its significance in protecting vulnerable populations. However, their support was less vocal compared to the governing parties. Alongside their endorsement of the campaign, they especially highlighted systematic weaknesses in the health sector and demanded higher wages for healthcare personnel, emphasizing the need for broader reforms in the healthcare system. In stark contrast, the Alternative for Germany (AfD) opposed the vaccination campaign. The AfD not only criticized the government’s approach but also spread fears about potential side effects of the vaccines. They claimed that the government was suppressing discussions about these side effects, fostering skepticism and resistance among certain segments of the population.

### Potential factors related to vaccine hesitancy

The literature has identified various factors associated with vaccine hesitation and parental intention to vaccinate their children, including socioeconomic status, demographic factors, and concerns about vaccine safety and effectiveness^45–48^. Table 1 shows descriptive statistics for the control variables used in the regressions. 45% of the parents in the sample are male, with an average age of 42. 58% work full-time, and the majority has a university-entrance diploma (Abitur). Given the large differences in willingness to vaccinate across the political spectrum^49^, we also account for party preferences. We include all control variables in our regressions because the computation of variance inflation factors does not indicate problematic multicollinearity (see Table A3 in the Appendix).

**Table 1:** Descriptive statistics.

Characteristics of the neighborhood		Avg.	SD	Min	Max
Days federal emergency brake (between 23 April to 30 June 2021)		13.61	6.92	0	39
Days with closed schools (between 23 April to 30 June 2021)		5.54	6.37	0	24
Migration share in city district		22.28	12.66	0	68.64
Unemployment rate city district		5.72	3.28	0	24.72
Characteristics of the parents					
Age		42.24	8.42	18	60
		**Share (in %)**			
Male		45			
Fulltime		58			
Educ.	Basic track or less	10			
	Intermediate	36			
	University-entrance diploma	53			
German		96			
Private health insurance		16			
Married		65			
Party	Christian democrats (CDU/CSU)	18			
	Social democrats (SPD)	19			
	Green party	15			
	Liberal party (FDP)	9			
	Left-wing (Die Linke)	7			
	Right-wing (AfD)	9			
	Other	21			
	Nonvoter	3			
Working from home (WFH) possible		45			
WFH	WFH not possible	55			
	Without restrictions	19			
	Only during wfh duty	13			
	With restrictions	9			
High building		9			
Share high school degree		35			

Smaragd Survey, Wave 3, January 2022. Number of observations: 1,819. Additional background information on each variable is provided in Table A2. The variable “Working from home (WFH) possible” includes the response to the question of whether WFH would be theoretically possible. The following WFH variables indicate the actual possibilities. High building refers to whether respondents live in a skyscraper.

**Fig. 2:**
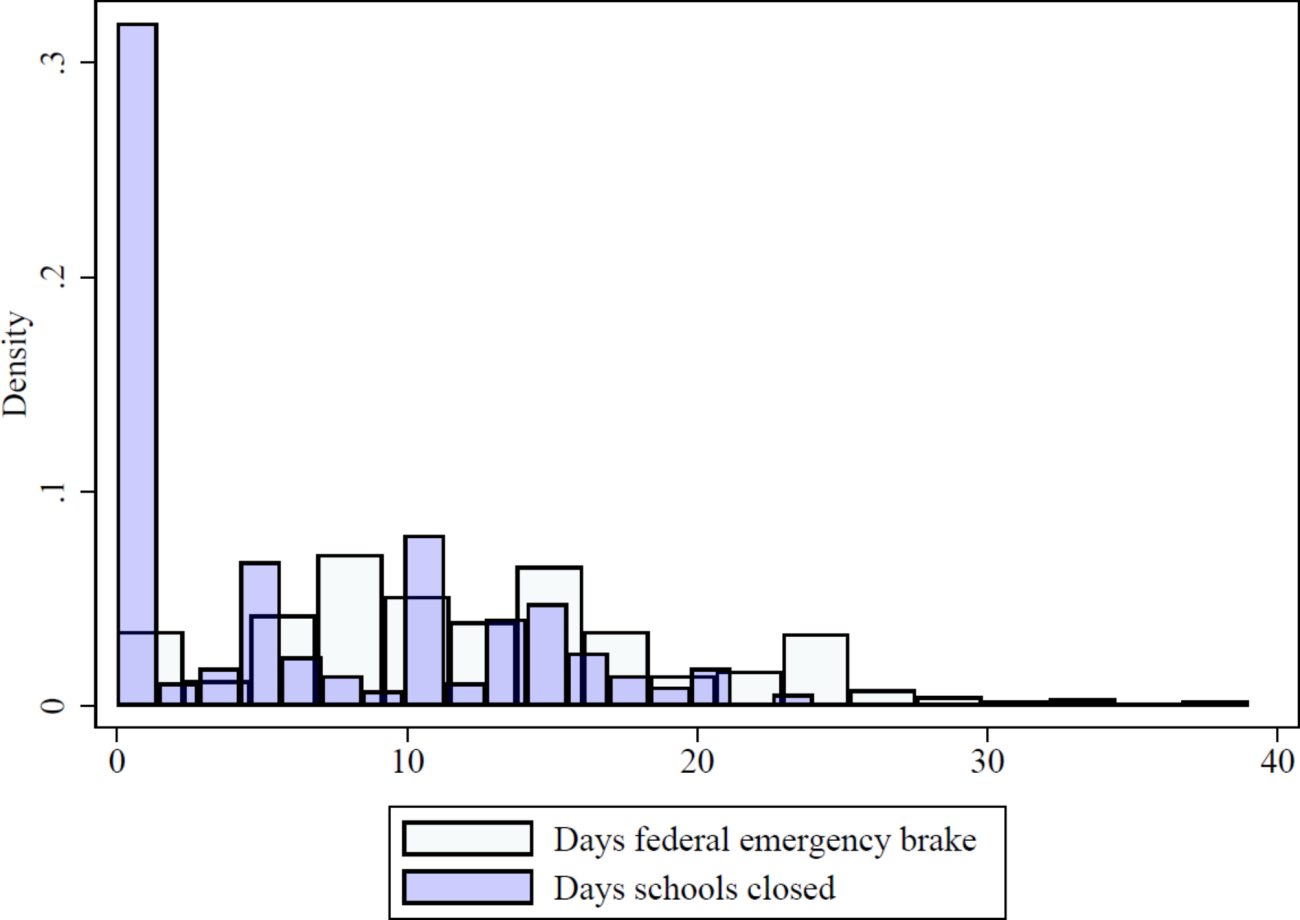
Distribution federal emergency brake and closed schools. Own calculation. The figure shows the number of days with an active federal emergency brake or closed schools between April 23 and June 30, 2021, when the federal emergency brake rules were in effect.

A potentially relevant characteristic of child vaccination behavior is its own (parental) negative experience with the coronavirus crisis. Specifically, we test whether parents who had stronger experience with restrictions due to the pandemic (e.g., because of school closures) are more willing to vaccinate themselves and their children. Figure 2 reports the distribution of days when the federal emergency brake (*Bundesnotbremse*) was put on in the county and when schools were closed. Starting in April 2021, the federal emergency brake became effective when the seven-day incidence in a county exceeded 100 per 100.000 people. It mainly included restrictions in gathering with people from other households, night-time curfews, requirements of a negative Corona test to shop, shutdowns of cultural activities, and regular tests and schooling only every second day. A seven-day incidence of more than 165 in three consecutive days led to school closures in the county.

Theoretically, there could be reverse causality between vaccination rates and the number of days with an active emergency brake, as higher vaccination rates could have successfully reduced the spread of the virus, resulting in fewer days with an active brake. However, we observe correlations that are very close to zero. There may be two reasons for this. First, the emergency brake was effective from April 2021, when vaccines were still scarce. Second, vaccines were ultimately more effective in reducing hospitalisations than in preventing infections.

### Statistical analysis

Depending on the nature of the outcome variables, we use different regression techniques. In our baseline regression, which are the characteristics of vaccination status, we run a linear probability model using OLS. Because of the binary nature of the outcome vaccination status, Logit or Probit regression would also be possible. However, in our application, the marginal effects after Logit and Probit are the same as the coefficients in the linear probability model. Thus, we choose the simplest model. Among those with children under 12, we take two approaches. First, we run the simple linear probability model and exclude parents who answer to be ‘not yet decided’. In addition, we estimate a multinomial logit regression where we include “yes”, “not yet decided” and “no” as separate unordered categories and report its marginal effects. Note here that one regression produces three sets of marginal effects (one per outcome category), with the effect of each variable summing to zero across all three categories and without imposing an order between these categories.

In order to increase the flexibility of the regression models, we also include interactions of relevant variables. We do this in the baseline specifications of Table 2 below. In particular, we want to allow for the possibility that persons with different experiences of Corona restrictions might have different effects of labor market participation and party preferences on vaccination behavior. Thus, in addition to the baseline variables, we also include interactions of days with federal emergency brakes (and days with closed schools) with the indicator of full time work and with all indicators of party preferences. For the sake of legibility and to keep the focus of our analysis, we do not discuss the interaction terms but calculate and report average marginal effects of all baseline variables.

The marginal effects are calculated as follows. Consider, as an example, the effect for fulltime work. Let us denote the outcome variable = Y, Fulltime = X1, Days federal emergency brake = X2, and Days with closed schools = X3. Then, the regression looks like Y = $\:{\beta\:}_{0}$ + $\:{\beta\:}_{1}$ X1 + $\:{\beta\:}_{2}$ X2 + $\:{\beta\:}_{3}$ X3 + $\:{\beta\:}_{4}$ X1*X2 + $\:{\beta\:}_{4}$ X1*X3 + …. The marginal effect of Fulltime for each person is then $\:{\beta\:}_{1}$ + $\:{\beta\:}_{4}$ X2 + $\:{\beta\:}_{5}$ X3, where person specific values of X2 and X3 are plugged in. Finally, the average of all individual marginal effects is reported. We used the *margins* command in Stata to calculate marginal effects and standard errors. Since the results with interactions are very similar to those without (reported in Table A4), we go on with the simpler model without interactions from Table 3 onwards.

Throughout the regression analysis, continuous variables are standardized while binary variables are not. Finally, note that this kind of analysis does not allow for any causal interpretations. Both, the variables on the left hand side and many of those on the right hand side of the regression equation are choice variables which are most likely affected by unobserved variables. Thus, variables like education or voting preferences are endogenous. Nevertheless, it is possible to interpret the results as associations which still allows to draw relevant conclusions.

## Results

### Characteristics of vaccination decision

Table 2 reports results of five linear regressions (linear probability model) of vaccination willingness of parents for (1) themselves, (2) and (3) children below 12, and (4) and (5) children aged 12–17. When child vaccination is the regression outcome, we run regressions with the same control variables as when the outcome is own vaccination, first without and then with own vaccination status as an additional control variable. This table includes marginal effects from regressions with interactions. Table A4 in the supplementary materials shows the results of the same regressions without any interactions. The results are very similar.

**Table 2:** Baseline regressions (average marginal effects).

		Own	Kids U12		Kids 12–17	
		(1)	(2)	(3)	(4)	(5)
Vaccinated (self)				0.536***		0.631***
				(0.052)		(0.035)
Days federal emergency brake		0.010	0.027	0.018	0.011	0.008
		(0.009)	(0.020)	(0.018)	(0.016)	(0.014)
Days with closed schools		0.025***	-0.007	-0.022	0.010	-0.009
		(0.010)	(0.022)	(0.021)	(0.017)	(0.015)
Male		0.041**	0.077**	0.088**	-0.062**	-0.104***
		(0.017)	(0.037)	(0.035)	(0.029)	(0.025)
Age		-0.021***	0.092***	0.092***	0.021*	0.038***
		(0.007)	(0.018)	(0.017)	(0.013)	(0.011)
Fulltime		0.015	0.063	0.022	0.069**	0.072***
		(0.017)	(0.040)	(0.038)	(0.029)	(0.025)
Educ.	Basic track or less	Reference				
	Intermediate	0.037*	0.183***	0.205***	0.016	-0.039
		(0.019)	(0.047)	(0.044)	(0.034)	(0.030)
	University-entrance diploma	0.011	0.156***	0.207***	0.051	0.005
		(0.021)	(0.048)	(0.045)	(0.037)	(0.032)
German		-0.046**	-0.012	0.032	0.065	0.088**
		(0.022)	(0.047)	(0.044)	(0.040)	(0.035)
Private health insurance		-0.079***	-0.080*	-0.032	-0.179***	-0.093***
		(0.021)	(0.041)	(0.039)	(0.037)	(0.033)
Married		0.050***	-0.001	0.000	0.036	-0.005
		(0.015)	(0.035)	(0.033)	(0.027)	(0.024)
Party	Christian democrats (CDU/CSU)	Reference				
	Social democrats (SPD)	0.054**	-0.117**	-0.104**	0.089**	0.016
		(0.024)	(0.053)	(0.050)	(0.043)	(0.038)
	Green party	-0.007	0.065	0.091*	0.078	0.069
		(0.026)	(0.057)	(0.053)	(0.050)	(0.043)
	Liberal party (FDP)	-0.031	-0.179**	-0.142**	0.048	0.056
		(0.031)	(0.072)	(0.067)	(0.056)	(0.049)
	Left-wing (Die Linke)	-0.152***	-0.287***	-0.170**	-0.084	0.054
		(0.036)	(0.076)	(0.071)	(0.072)	(0.064)
	Right-wing (AfD)	-0.238***	-0.694***	-0.477***	-0.132***	0.005
		(0.027)	(0.060)	(0.059)	(0.047)	(0.042)
	Other	-0.030	-0.336***	-0.292***	-0.031	-0.034
		(0.022)	(0.051)	(0.048)	(0.039)	(0.034)
	Nonvoter	-0.314***	-0.448***	-0.308***	-0.166***	0.012
		(0.034)	(0.096)	(0.090)	(0.057)	(0.051)
Working from home (WFH) possible		0.202***	-0.021	-0.093	0.148**	-0.030
		(0.036)	(0.094)	(0.088)	(0.061)	(0.054)
WFH	not possible	Reference				
	Without restrictions	-0.149***	-0.039	-0.002	-0.146**	-0.013
		(0.039)	(0.099)	(0.092)	(0.067)	(0.059)
	Only during wfh duty	-0.115***	0.055	0.096	-0.160**	-0.044
		(0.041)	(0.104)	(0.097)	(0.070)	(0.062)
	With restrictions	-0.136***	0.102	0.161	-0.055	0.080
		(0.044)	(0.109)	(0.102)	(0.079)	(0.069)
High building		0.089***	0.075	0.050	0.111*	0.104**
		(0.031)	(0.073)	(0.069)	(0.059)	(0.052)
Migration share in city district		-0.009	0.021	-0.006	-0.069***	-0.057***
		(0.012)	(0.027)	(0.025)	(0.022)	(0.019)
Unemployment rate city district		-0.050***	-0.048**	-0.005	-0.051***	-0.024
		(0.010)	(0.022)	(0.021)	(0.017)	(0.015)
Share high school degree		-0.035***	0.008	0.021	-0.030*	-0.005
		(0.010)	(0.020)	(0.019)	(0.018)	(0.016)
Federal state binary indicators		yes	yes	yes	yes	yes
Observations		1817	798	798	1145	1145
F-Statistic		15.18	10.43	13.69	11.18	20.13

Smaragd Survey, Wave 3/January 2022. Weighted regressions. Average marginal effects reported. Standard errors are in parentheses; *(*p* < 0.1), ** (*p* < 0.05), *** (*p* < 0.01). Continuous variables are standardized. The regression model includes interactions of days with federal emergency brakes (and days with closed schools) with the indicator of fulltime work and with all indicators of party preferences. Average marginal effects are reported. The variable “Working from home (WFH) possible” includes the response to the question of whether WFH would be theoretically possible. The following WFH variables indicate the actual possibilities. High building refers to whether respondents live in a skyscraper.

Our results in column (1) show which characteristics are associated with own vaccination status. Since about 90% of the sample is already vaccinated, there is not much variation to exploit in our regression. This is likely the reason why not many coefficients are significant in column (1). Nevertheless, we observe a strong correlation between party affiliation and vaccination status. Voters of both political extremes, although to a lesser extent for the left, and non-voters are less likely to be vaccinated. Since the continuous control variables are standardized, we can directly interpret their relative size. Here we find that the surrounding unemployment rate has the largest correlation, i.e. individuals in city districts with higher unemployment rates – implying worse socio-economic status of the area – are less likely to be vaccinated.

In the following columns we turn to the associations with childhood vaccinations, separately for those under 12 and over 12 years of age. We repeat these regressions with and without parental vaccination status as an additional control variable. Including this variable leads to very little difference in the marginal effects of vaccinating children under 12, but many marginal effects of vaccinating children over 12 lose their significance. This is probably due to the high and congruent vaccination rates of parents (around 90%) and older children (almost 80%). Because both are highly correlated, inclusion of own vaccination largely explains adolescent vaccination. Thus, there is very little variation left when parents’ own vaccination status is added. This is different for child vaccination where the vaccination rates are much lower and the correlation with own vaccination is still there but less pronounced. Yet, own vaccination turns out to be a strong predictor, we focus our interpretation below on columns (3) and (5).

Columns (3) and (5) show that vaccinated parents are 54 and 63% points more likely to vaccinate their children under 12 and over 12, respectively. Apart from this relationship, the largest associations of vaccination status of children under 12 years of age are with party affiliation. Here, Green voters are significantly more likely to vaccinate their children compared to conservative voters, while the opposite is true for voters of the Social Democrats, the Liberals and the radical Left – but especially for voters of other (smaller) parties, non-voters and of the extreme right. In addition, parents with higher levels of education are also more likely to vaccinate their children. We also observe that fathers are much more likely to report willingness to have their children under 12 vaccinated than mothers. This is in line with much of the literature cited in the Introduction. Yet, note again, that this does not necessarily result in actual vaccination as this is likely a joint decision of both parents which we do not observe.

For children aged 12 and over, the characteristics of parents who choose to vaccinate show some notable differences compared with those for younger children. A significant predictor of vaccination for this age group is whether the parent works full-time, probably due to concerns that a sick child would require time off work. In contrast to the findings for younger children, educational differences no longer play a significant role in vaccination decisions. However, German nationality is associated with a higher likelihood of vaccinating older children, which is accompanied by lower vaccination rates in neighbourhoods with a higher proportion of foreign residents. In addition, parents living in high-rise buildings are more likely to vaccinate their children, while those with private health insurance are less likely to do so.

Notably, party affiliation loses its statistical significance in this context, possibly due to the strong correlation between parents’ own vaccination status and their political preferences. This is particularly evident among AfD voters, who have significantly lower vaccination rates. Given the high overlap between parental vaccination (90%) and vaccination of children over 12 years of age (80%) mentioned above, this congruence is also likely to reduce the independent effect of party preference on vaccination decisions.

Interestingly, there is a reversal in the association with parental gender, with fathers now less likely than mothers to vaccinate their older children, although caution should be exercised in over-interpreting this shift, as discussed in the interpretation of the results for vaccination decisions for children under 12. The age coefficient remains positive, although smaller, suggesting that older parents are still more likely to vaccinate their children, possibly influenced by the older age of these children.

As some of the variables included in our regressions are clearly correlated, multicollinearity may pose a potential issue. Table A3 reports the variance inflation factors after the regressions in Table 2. As a rule of thumb, a variable should not have a variance inflation factor of greater than 10. Because they are all less than 10, we argue that multicollinearity is not an issue in this application.

### Vaccinate yourself but not your children?

**Fig. 3:**
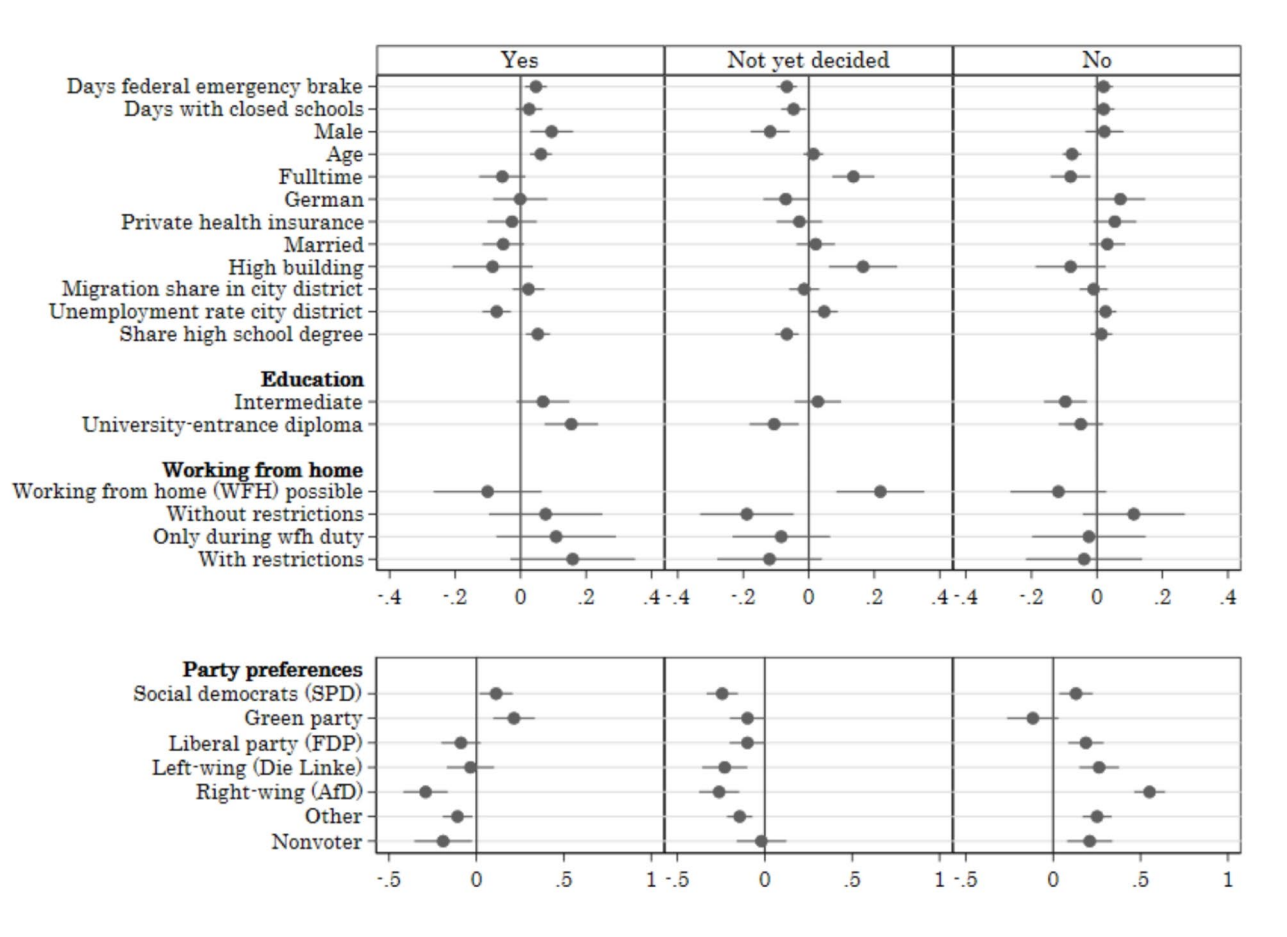
Multinomial logit, marginal effects. Smart survey, Wave 3, January 2022. Average marginal effects after weighted multinomial logit regressions. The outcome variable is willingness to vaccinate children aged between 5 and 11 years. The sample only includes parents who are vaccinated and have children aged between 5 and 11 years. Standard errors are in parentheses; * (*p* < 0.1), ** (*p* < 0.05), *** (*p* < 0.01). Continuous variables are standardized. The marginal effects are also reported in Table A5 in the appendix.

Next, we restrict the sample to parents who are vaccinated, and ask the question: Who vaccinates themselves but not their children? Given that most teenagers aged between 12 and 17 years are already vaccinated (in particular among the vaccinated parents), we restrict this analysis to vaccination of children aged 5–11. Here, we distinguish three groups, *yes*, *not yet decided*, and n*o*, by running a multinomial logit regression with these three outcome categories without imposing any order and with the same control variables as before. In Fig. 3, we report the marginal effects of the multinomial logit regression. Note that one regression produces three sets of marginal effects (one per outcome category), where the effects of each variable sum to zero across all three categories. The regression table which includes the same marginal effects is reported in Table A5 in the Appendix. Note that both parts of Fig. 3 are from the same regression. However, to improve readability, we present the marginal effects on two different scales. This is because all effects are dominated by the large magnitude of the effects of party preferences.

The associations between party preferences and child vaccination status are consistent with the findings above. Parents who vote for the Green Party are persistently more likely to have vaccinated their child and less likely to have not vaccinated their child. On the contrary, extreme right voters are less likely to have vaccinated and more likely to have not vaccinated their children. In fact, the decision not to vaccinate their children is statistically significantly higher than for any other partisan group. Another important characteristic is the school education of the parents. Parents with the highest level of school education are more likely to vaccinate their children. The relationships are less clear for other socio-demographic characteristics, such as labor force participation or marital status.

**Fig. 4:**
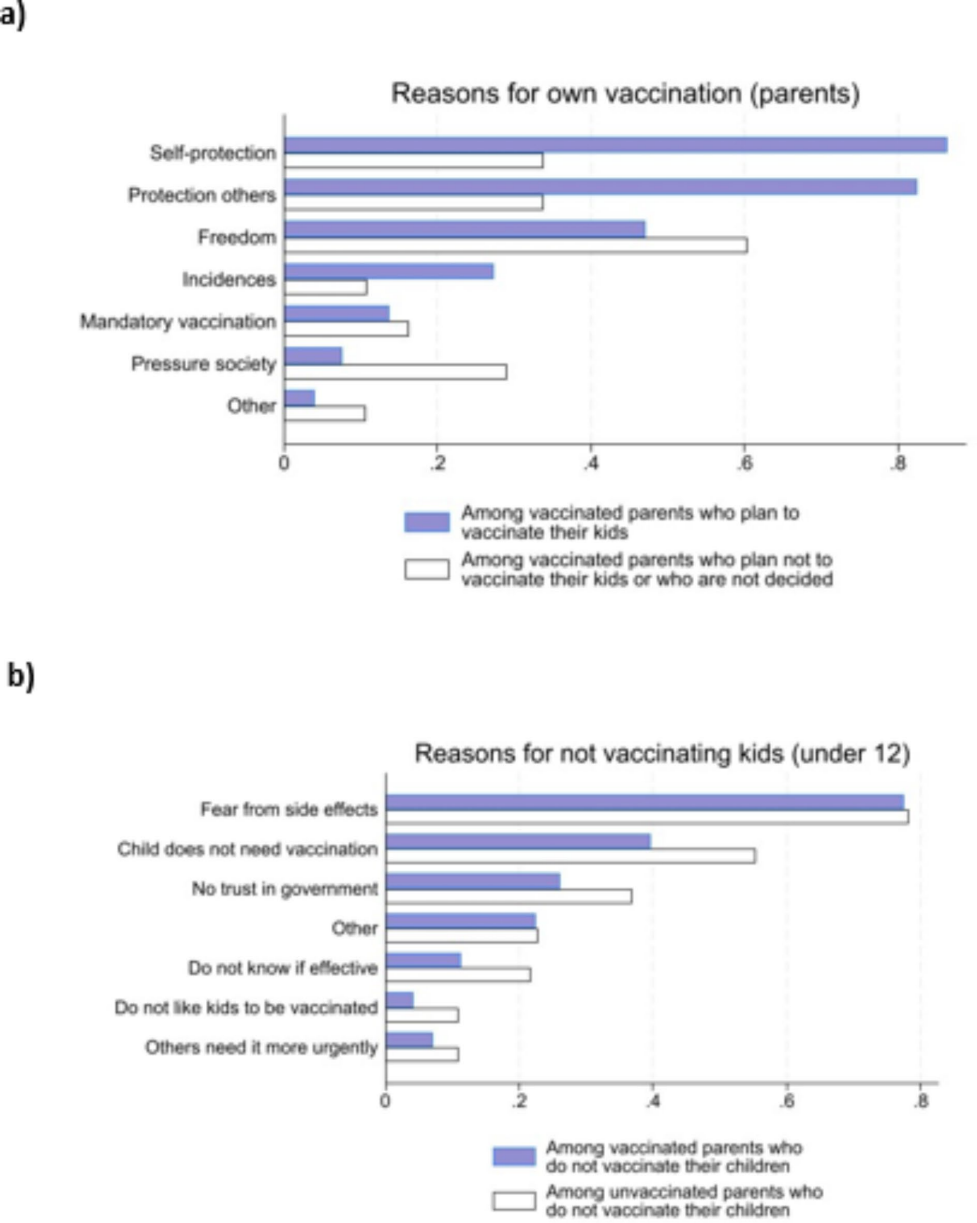
Reasons for and against vaccination. The sample for panel (**a**) only includes parents who are vaccinated and have children between 5 and 11. The sample for panel (**b**) only includes parents who do not plan to vaccinate their 5–11 year old children. Respondents were able to check for several possible reasons. Answers left panel: Self-protection = For own health protection; Protection others = Protection of others or Because I have high-risk patients in my environment; Freedom = Because vaccinated people have more freedom or because vaccination saves me tests; mandatory vaccination = I would like to get ahead of the possible vaccination requirement; Pressure society = Out of social pressure; Incidence = The high Incidences = The led me to vaccinate.

Next, we analyze the stated reasons for and against vaccination. This is illustrated in Fig. 4. Parents who state that they are vaccinated are asked about their reasons for vaccination. Likewise, parents who state that they are not willing to vaccinate their children are asked about their reasons. Panel (a) on the left takes the perspective of the parents by providing their reasons for their own vaccination. Multiple responses were possible. We show the results grouped by parents who plan to vaccinate their own 5–11 year old children (dark bars) versus those who do not (white bars). The most important reason for own vaccination is the protection of others. More than 80% of the group that also planned to vaccinate their children (dark bars) mention this reason. Only 48% in this group vaccinate themselves to get more rights and personal freedoms (e.g., be able to attend cultural events that, at that time, usually required vaccination). In contrast, parents who do not plan to vaccinate their own children (white bars) only have a 30–35% likelihood of vaccinating themselves because of their own protection or protection of others. Instead, 60% state that they should vaccinate themselves to achieve more freedom. Moreover, 30% of these parents plan to vaccinate due to high societal pressure (less than 10% of those who plan to vaccinate their children). This shows that those who do not vaccinate their children put a smaller weight on health reasons and a higher weight on personal freedom and other reasons when they decide about their own vaccination.

Panel (b) of Fig. 4 changes the perspective and looks at reasons *not* vaccinating children. Now, only parents who do not plan to vaccinate their children are in the sample, and the groups are split by parental vaccination status. The dark bars represent parents who vaccinate themselves but not their children, whereas the white bars represent parents who neither vaccinate themselves nor their children. One outstanding reason not to vaccinate children is the fear of side effects, which accounts for almost 80%. This seems to be unrelated to willingness to receive vaccination themselves. However, parents who are not vaccinated themselves state additional reasons for vaccination with a higher likelihood than vaccinated parents. More than 50% believe that their children do not need vaccination (because the Corona virus is not a serious health threat for children), while almost 40% also mention that they do not trust the government in general.

### Parental characteristics by reasons not to vaccinate

Table 3 displays the factors that affect the decision of some parents to not vaccinate their 5–11 years old children. We conducted regressions, featured in columns (1) and (3), on a sample consisting of parents of children aged between 5 and 11 who report that they do not vaccinate their children. These parents state the reasons for their decision, as shown in Fig. 4 (b). We estimate linear probability models, where the outcome variable is a binary indicator for *fear of side effects* (1) *or no trust in the government* (3). In columns (2) and (4), we enrich the sample. We argue that parents who have already vaccinated their children have no (strong) fear of side effects and trust in the government (at least regarding Corona vaccination). Thus, we add the group of parents who state to vaccinate their children between 5 and 11, coding the outcome variables as zero.

**Table 3:** Characteristics of reasons not to vaccinate.

		Fear of Side Effects		No trust in government	
		(1)	(2)	(3)	(4)
Male		-0.239***	-0.082**	0.004	-0.022
		(0.062)	(0.035)	(0.073)	(0.027)
Age		0.056**	-0.067***	0.056*	-0.009
		(0.027)	(0.017)	(0.031)	(0.013)
Fulltime		0.075	-0.052	0.057	0.007
		(0.062)	(0.039)	(0.072)	(0.030)
Educ.	Basic track or less	Reference			
	Intermediate	-0.348***	-0.262***	-0.022	-0.023
		(0.063)	(0.044)	(0.074)	(0.033)
	University-entrance diploma	-0.298***	-0.249***	0.017	-0.039
		(0.068)	(0.045)	(0.079)	(0.034)
German		-0.054	0.012	0.226**	0.111***
		(0.078)	(0.043)	(0.091)	(0.033)
Private health insurance		0.172**	0.141***	0.201***	0.095***
		(0.066)	(0.039)	(0.077)	(0.030)
Married		0.148***	0.067**	0.004	0.033
		(0.053)	(0.034)	(0.062)	(0.026)
Party	Christian democrats (CDU/CSU)	Reference			
	Social democrats (SPD)	0.030	0.062	-0.072	0.034
		(0.105)	(0.050)	(0.122)	(0.038)
	Green party	-0.530***	-0.093*	-0.268	0.004
		(0.181)	(0.055)	(0.210)	(0.041)
	Liberal party (FDP)	0.128	0.205***	0.352***	0.235***
		(0.107)	(0.063)	(0.125)	(0.048)
	Left-wing (Die Linke)	0.205	0.292***	0.110	0.210***
		(0.135)	(0.072)	(0.157)	(0.055)
	Right-wing (AfD)	0.346***	0.695***	0.422***	0.527***
		(0.098)	(0.056)	(0.114)	(0.042)
	Other	0.083	0.215***	0.073	0.123***
		(0.090)	(0.048)	(0.105)	(0.036)
	Nonvoter	-0.044	0.093	0.035	0.170**
		(0.122)	(0.088)	(0.142)	(0.067)
Working from home (WFH) possible		-0.060	-0.088	-0.089	-0.115*
		(0.114)	(0.091)	(0.132)	(0.069)
WFH	not possible	Reference			
	Without restrictions	0.025	0.082	0.471***	0.152**
		(0.132)	(0.096)	(0.153)	(0.073)
	Only during wfh duty	0.055	0.061	-0.064	0.073
		(0.158)	(0.102)	(0.183)	(0.078)
	With restrictions	-0.021	-0.007	-0.119	0.041
		(0.157)	(0.106)	(0.183)	(0.081)
High building		0.131	-0.016	-0.279**	-0.139***
		(0.107)	(0.071)	(0.124)	(0.054)
Migration share in city district		0.073*	-0.034	-0.054	-0.068***
		(0.041)	(0.025)	(0.048)	(0.019)
Unemployment rate city district		-0.059**	0.041**	0.085***	0.081***
		(0.027)	(0.020)	(0.031)	(0.015)
Share high school degree		-0.003	0.026	0.081**	0.071***
		(0.029)	(0.017)	(0.034)	(0.013)
Federal state binary indicators		yes	yes	yes	yes
Observations		288	762	288	762
F-Statistic		5.562	12.271	8.583	14.583

Smaragd survey, Wave 3, January 2022. Columns (1) and (3) only include parents who state that they do not vaccinate their 5–11 year old children or are undecided. Columns (2) and (4) include all the parents of 5–11 year old children. The standard errors are shown in parentheses. * (*p* < 0.1), ** (*p* < 0.05), *** (*p* < 0.01). Continuous variables are standardized. The variable “Working from home (WFH) possible” includes the response to the question of whether WFH would be theoretically possible. The following WFH variables indicate the actual possibilities. High building refers to whether respondents live in a skyscraper.

As a result, most of the variation can be attributed to party preferences. AfD voters are more likely to report both a fear of side effects and a lack of trust in the government. To a lesser extent, all other voters, with the exception of Green voters, are (slightly) more likely to report such fears compared to the conservatives. Greens, by contrast, report statistically significantly less fear of side effects. Fear of side effects is also less common among men and the better educated, and more common among married people and those with private health insurance. Lack of trust in the government is more common among Germans, privately insured parents and those who can work remotely without restrictions. A higher unemployment rate in the urban area and a higher proportion of neighbors with a high school degree are also associated with a lack of trust in the government. Conversely, parents living in high-rise buildings and in neighborhoods with a higher proportion of immigrants are less likely to report a lack of trust in government.

## Discussion and conclusion

Our study aims to identify the socioeconomic and ecological characteristics of parents’ willingness to vaccinate themselves and their children against COVID-19. We pay particular attention to parents who choose to vaccinate themselves but not their children, in particular children younger than 12 years. We analyze the reasons for not vaccinating their children and the socioeconomic characteristics of parents that correlate with this decision.

Our main findings indicate that a significant proportion of parents opt to vaccinate themselves but not their children. This trend is particularly pronounced among individuals who belong to one of the two political extremes, and is even more pronounced among far-right voters. In addition, the same pattern is observed among parents with lower levels of education. This finding is in line with findings for other countries [51, 52, 16]. Moreover, and in general, districts with worse socio-economic status have a lower share of vaccinated children. Fear of side effects is overwhelmingly the most common reason cited by parents for not vaccinating their children. This finding is also comparable to findings in other studies [51, 52]. This fear is most pronounced among parents with lower education levels, women [54] as well as those with left-wing or right-wing political affiliations. Our findings support those of previous studies on adult vaccination. Protecting oneself and others from disease is a primary motivation for vaccination, although concerns about vaccine safety remain a major argument^12,20,50^. The mainstream media often reported on the safety of the coronavirus vaccination, but these messages failed to reach low-educated individuals and those at the political extremes, or these groups remained skeptical.

When vaccination campaigns are designed in the future, public policy should pay special attention to individuals living in disadvantaged areas with higher unemployment rates, higher shares of migrants and voters for more extreme parties. Given the comparably large adult vaccination rates, campaigns could be more specifically tailored to child vaccinations. Luckily, these areas can be identified using official data. That is, campaigns that both make aware and inform but also make child vaccination more accessible in these districts (like mobile vaccination teams that enter these areas) might help to reach the goal of higher child vaccination rates.

### Limitations

This study has several limitations. First, this is an online survey with a relatively small sample size. We show how it compares to administrative data for the full German population in terms of important observable characteristics, but we cannot guarantee that it is representative of unobservable characteristics. In particular, we cannot account for those who did not participate in the survey. Second, we cannot entirely rule out the possibility that our sample still includes parents of children under five years of age, although this should be a small number. Moreover, while actual vaccination status was asked for children and adults above 12 years, we only had information on the stated willingness to vaccinate children younger than 12 years. Third, information on partners is missing as well as the exact number of children in the relevant age groups. Fourth and most importantly, we can only establish associations and cannot make causal statements.

These associations may be biased due to the nature of data collection. We rely on voluntary participation in the online survey. Hence, there may be both selection and response bias. Participants in such surveys may have a greater interest in politics and the pandemic, resulting in varying perspectives on vaccination decisions. However, the generalizability of our findings is limited by these biases. However, the validity of our findings is supported by their consistency with the results of other studies.

## Electronic supplementary material

Below is the link to the electronic supplementary material.

Supplementary Material 1

## Data Availability

Data cannot be shared publicly because of their proprietary nature - Copy Right belongs to the market research institute infas360. Data are available from FDZ Ruhr (contact via fdz@rwi-essen.de) for researchers who meet the criteria for access to confidential data. Access is limited to a Secure Room at the FDZ, and its use is limited to the purposes of replication.

## References

[1] Kelvin, A. A. & Halperin, S. COVID-19 in children: the link in the transmission chain. *Lancet*. **20**, 633–634 (2020). 10.1016/S1473-3099(20)30236-X PMC7156154 32220651

[2] Jones, T. C. et al. Estimating infectiousness throughout SARS-CoV-2 infection course. *Science*. **373**, eabi5273 (2021). 10.1126/science.abi5273 PMC9267347 34035154

[3] Ludvigsson, J. F. Children are unlikely to be the main drivers of the COVID-19 pandemic - A systematic review. *Acta Paediatr. ***109** (8), 1525–1530 (2020). 10.1111/apa.15371 PMC7280674 32430964

[4] Li, Y. et al. The temporal association of introducing and lifting non-pharmaceutical interventions with the time-varying reproduction number (R) of SARS-CoV-2: a modelling study across 131 countries. *Lancet*. **21**, 193–202 (2021). 10.1016/S1473-3099(20)30785-4 PMC7581351 33729915

[5] Flaxman, S. et al. Eaton and others, estimating the effects of non-pharmaceutical interventions on COVID-19 in Europe. *Nature*. **584**, 257–261 (2020). 32512579 10.1038/s41586-020-2405-7

[6] Yang, J. et al. COVID-19 vaccination in Chinese children: a cross-sectional study on the cognition, psychological anxiety state and the willingness toward vaccination. *Human Vaccines Immunotherapeutics*. **18**, 1–7 (2021). 34324407 10.1080/21645515.2021.1949950 PMC8920157

[7] Isphording, I. E., Lipfert, M. & Pestel, N. Does re-opening schools contribute to the spread of SARS-CoV-2? Evidence from staggered summer breaks in Germany. *J. Public. Econ. ***198**, 104426 (2021).

[8] Von Bismarck-Osten, C., Borusyak, K. & Schönberg, U. The role of schools in transmission of the SARS-CoV-2 virus: quasi-experimental evidence from Germany. *Econ. Pol*. **37**, 87–130 (2022).

[9] Levinson, M., Cevik, M. & Lipsitch, M. Reopening primary schools during the pandemic. *N. Engl. J. Med. ***383**, 981–985 (2020). 32726550 10.1056/NEJMms2024920

[10] Statista Impfquote gegen das Coronavirus (COVID-19) in Deutschland nach Altersgruppen (Stand: 2. Januar 2023), (2023).

[11] Jørgensen, F. J. & Petersen, M. B. *Considerations Underlying Parents’ Acceptance of COVID-19 Vaccines for Their Child: Evidence from Denmark*, PsyArXiv, (2021).

[12] Bell, S., Clarke, R., Mounier-Jack, S., Walker, J. L. & Paterson, P. Parents’ and guardians’ views on the acceptability of a future COVID-19 vaccine: A multi-methods study in England. *Vaccine*. **8**, 7789–7798 (2020). 10.1016/j.vaccine.2020.10.027 PMC7569401 33109389

[13] Brandstetter, S. et al. U. N. O.-K. study group, parents’ intention to get vaccinated and to have their child vaccinated against COVID-19: cross-sectional analyses using data from the KUNO-Kids health study. *Eur. J. Pediatrics*. **180**, 3405–3410 (2021). 10.1007/s00431-021-04094-z PMC8127511 33999257

[14] Rees, F. et al. Measuring parents’ readiness to vaccinate themselves and their children against COVID-19. *Vaccine*. **40**, 3825–3834 (2022). 10.1016/j.vaccine.2022.04.091 PMC9069251 35623906

[15] Pierantoni, L. et al. Nationwide COVID-19 survey of Italian parents reveals useful information on attitudes to school attendance, medical support, vaccines and drug trials. *Acta Paediatr. ***110**, 942–943 (2021). 33047328 10.1111/apa.15614 PMC7677859

[16] Montalti, M. et al. Would Parents Get Their Children Vaccinated Against SARS-CoV-2? Rate and Predictors of Vaccine Hesitancy According to a Survey over 5000 Families from Bologna, Italy. *Vaccines*. **9**, (2021). 10.3390/vaccines9040366 PMC8069076 33920109

[17] Giuseppe, G. D., Pelullo, C. P., Volgare, A. S., Napolitano, F. & Pavia, M. Parents’ willingness to Vaccinate their children with COVID-19 vaccine: results of a survey in Italy. *J. Adolesc. Health*. **70**, 550–558 (2022). 35305792 10.1016/j.jadohealth.2022.01.003 PMC8767903

[18] Lecce, M. et al. May, Caregivers’ Intention to Vaccinate Their Children Under 12 Years of Age Against COVID-19: A Cross-Sectional Multi-Center Study in Milan, Italy. *Frontiers Pediatrics*, **10**, 834363 (2022). 10.3389/fped.2022.834363 PMC9196897 35712618

[19] Babicki, M., Pokorna-Kałwak, D., Doniec, Z. & Mastalerz-Migas, A. Attitudes of Parents with Regard to Vaccination of Children against COVID-19 in Poland. A Nationwide Online Survey. *Vaccines*. **9**, 1192, (2021). 10.3390/vaccines9101192 PMC8539339 34696300

[20] Goldman, R. D. et al. Caregiver willingness to vaccinate their children against COVID-19: Cross sectional survey. *Vaccine*. **38**, 7668–7673 (2020). 10.1016/j.vaccine.2020.09.084 PMC7547568 33071002

[21] Nguyen, K. H., Nguyen, K., Geddes, M., Allen, J. D. & Corlin, L. Trends in adolescent COVID-19 vaccination receipt and parental intent to vaccinate their adolescent children, United States, July to October, 2021. *Ann. Med. ***54**, 733–742 (2022). 35238263 10.1080/07853890.2022.2045034 PMC8903754

[22] Allen, J. D. et al. Parents’ willingness to Vaccinate Children for COVID-19: conspiracy theories, information sources, and Perceived responsibility. *J. Health Communication*. **28**, 15–27 (2023). 36755480 10.1080/10810730.2023.2172107 PMC10038916

[23] Galanis, P. et al. Willingness, refusal and influential factors of parents to vaccinate their children against the COVID-19: a systematic review and meta-analysis. *Prev. Med. ***157**, 106994 (2022). 35183597 10.1016/j.ypmed.2022.106994 PMC8861629

[24] He, K., Mack, W. J., Neely, M., Lewis, L. & Anand, V. Parental perspectives on Immunizations: impact of the COVID-19 pandemic on Childhood Vaccine Hesitancy. *J. Community Health*. **47**, 39–52 (2021). 34297272 10.1007/s10900-021-01017-9 PMC8299444

[25] Skjefte, M. et al. COVID-19 Vaccine Acceptance Among Pregnant Women and Mothers of Young Children: Results of a Survey in 16 Countries. *Eur. J. Epidemiol*. **36**, 197–211 (2021). 10.1007/s10654-021-00728-6 PMC7920402 33649879

[26] Kelly, B. J. et al. Predictors of willingness to get a COVID-19 vaccine in the U.S. *BMC Infect. Dis. ***21**, 338 (2021). 33845781 10.1186/s12879-021-06023-9 PMC8039496

[27] Yılmaz, M. & Sahin, M. Parents’ willingness and attitudes concerning the COVID-19 vaccine: A cross-sectional study, *Inter. J. Clini. Prac*. **75**, (2021). 10.1111/ijcp.14364 PMC8236907 33998108

[28] Xu, Y. et al. Parental psychological distress and attitudes towards COVID-19 vaccination: a cross-sectional survey in Shenzhen, China. *J. Affect. Disord. ***292**, 552–558 (2021). 34147967 10.1016/j.jad.2021.06.003 PMC8179837

[29] Scherer, A. et al. Acceptability of Adolescent COVID-19 Vaccination Among Adolescents and Parents of Adolescents — United States, April 15–23, 2021, *MMWR. Morbidity and Mortality Weekly Report*. **70**, (2021). 10.15585/mmwr.mm7028e1 PMC8314712 34264908

[30] Teasdale, C. A. et al. Lans to Vaccinate Children for Coronavirus Disease 2019: a Survey of United States parents. *J. Pediatr. ***237**, 292–297 (2021). 34284035 10.1016/j.jpeds.2021.07.021 PMC8286233

[31] Dror, A. et al. Vaccine Hesitancy: The Next Challenge in the Fight Against COVID-19, *Eur. J. Epidemiol*. **35**, 775–779 (2020). 10.1007/s10654-020-00671-y PMC8851308 32785815

[32] Wang, Q. et al. Vaccine Hesitancy: COVID-19 and Influenza Vaccine Willingness among Parents in Wuxi, China—A Cross-Sectional Study. *Vaccines*. **9**, (2021). 10.3390/vaccines9040342 PMC8066309 33916277

[33] Hetherington, E. et al. SARS-CoV-2 vaccination intentions among mothers of children aged 9 to 12 years: a survey of the all our families cohort. *Can. Med. Association Open. Access. J. ***9**, E548–E555 (2021). 10.9778/cmajo.20200302 PMC8177949 34021012

[34] Bagateli, L. E. et al. COVID-19 Vaccine Hesitancy among Parents of Children and Adolescents Living in Brazil. *Vaccines*. **9**, (2021). 10.3390/vaccines9101115 PMC8540804 34696223

[35] Akarsu, B. et al. While studies on COVID-19 vaccine is ongoing, the public’s thoughts and attitudes to the future COVID-19 vaccine. *Int. J. Clin. Pract. ***75**, e13891 (2021). 33278857 10.1111/ijcp.13891 PMC7883065

[36] Aldakhil, H., Albedah, N., Alturaiki, N., Alajlan, R. & Abusalih, H. Vaccine hesitancy towards childhood immunizations as a predictor of mothers’ intention to vaccinate their children against COVID-19 in Saudi Arabia. *J. Infect. Public Health*. **14**, 1497–1504 (2021). 34481723 10.1016/j.jiph.2021.08.028 PMC8390407

[37] Temsah, M. H. et al. O. Temsah and others, parental attitudes and hesitancy about COVID-19 vs. routine childhood vaccinations: A National Survey. *Front Public. Health*. **9**, 752323 (2021). 10.3389/fpubh.2021.752323 PMC8548678 34722451

[38] Yigit, M., Ozkaya-Parlakay, A. & Senel, E. Evaluation of COVID-19 Vaccine Refusal in Parents. *Pediatr. Infect. Disease J. ***40**, e134–e136 (2021). 10.1097/INF.0000000000003042 33410650

[39] Zhou, Y., Zhang, J., Wu, W., Liang, M. & Wu, Q. S. Willingness to receive future COVID-19 vaccines following the COVID-19 epidemic in Shanghai, China. *BMC Public. Health*. **21**, 1103 (2021). 34107930 10.1186/s12889-021-11174-0 PMC8188944

[40] Nguyen, K. H., Nguyen, K., Mansfield, K., Allen, J. D. & Corlin, L. Child and adolescent COVID-19 vaccination status and reasons for non-vaccination by parental vaccination status. *Public. Health*. **209**, 82–89 (2022). 35870290 10.1016/j.puhe.2022.06.002 PMC9189141

[41] Rane, M. S. et al. Intention to Vaccinate Children against COVID-19 among Vaccinated and unvaccinated US parents. *JAMA Pediatr. ***176**, 201–203 (2022). 10.1001/jamapediatrics.2021.5153 PMC8649908 34870702

[42] infas 360, *Casa Monitor.*

[43] Hörnig, L. & Schaffner, S. FDZ data description, (2023).

[44] Heerwig, J. A. & McCabe, B. J. Education and social desirability bias: the case of a black presidential candidate. *Soc. Sci. Q. ***90**, 674–686 (2009).

[45] Nomura, S. et al. and others, Reasons for being unsure or unwilling regarding intention to take COVID-19 vaccine among Japanese people: A large cross-sectional national survey. *The Lancet Regional Health–Western Pacific*. **14**, (2021). 10.1016/j.lanwpc.2021.100223 PMC8324415 34368797

[46] Robinson, E., Jones, A. & Daly, M. International estimates of intended uptake and refusal of COVID-19 vaccines: A rapid systematic review and meta-analysis of large nationally representative samples. *Vaccine*, **39**, 2024–2034 (2021). 10.1016/j.vaccine.2021.02.005 PMC7867398 33722411

[47] Lin, C., Tu, P. & Beitsch, L. M. Confidence and receptivity for COVID-19 vaccines: a rapid systematic review, *Vaccines*. **9**, 16 (2020). 10.3390/vaccines9010016 PMC7823859 33396832

[48] Horiuchi, S. et al. and others, Factors of parental COVID-19 vaccine hesitancy: A cross sectional study in Japan, *PloS one*. **16**, e0261121 (2021). 10.1371/journal.pone.0261121 PMC8683027 34919580

[49] Wallace, J., Goldsmith-Pinkham, P. & Schwartz, J. L. Excess Death Rates for Republicans and Democrats During the COVID-19 Pandemic. (2022).

[50] Pan, F. et al. Parents’ Decisions to Vaccinate Children against COVID-19: A Scoping Review. *Vaccines*. **9**, 1479 (2021). 10.3390/vaccines9121476 PMC8705627 34960221

