# Supplementary material for: The individual and ecological characteristics of parental COVID-19 vaccination decisions
Source: Sci Rep. 2024 Oct 15;14:24194. doi: 10.1038/s41598-024-74963-8 (PMC11480482; doi:10.1038/s41598-024-74963-8)
Supplement: Supplementary file 1 — Supplementary Material 1 [file 41598_2024_74963_MOESM1_ESM.docx]

# Appendix

**Table A1 Representativeness of the Smaragd Survey compared to Administrative Data**

| Variable | Mean Casa Monitor | Administrative Data |
| --- | --- | --- |
| Age <30 | 0.17 | 0.15^a^ |
| 30 ≤ Age < 50 | 0.29 | 0.25^a^ |
| 50 ≤Age < 70 | 0.33 | 0.29^a^ |
| 70 ≤Age | 0.21 | 0.15^a^ |
| Male | 0.49 | 0.49^b^ |
| Household size^1^ | 2.39 | 2.02^b^ |
| Fulltime work | 0.41 | 0.35^a^ |
| German | 0.87 | 0.85^b^ |
| Married | 0.5 | 0.49^b^ |
| Private health insurance | 0.13 | 0.10^c^ |
| Basic track or less reference^2^ | 0.35 | 0.34^a^ |
| Intermediate^2^ | 0.31 | 0.31^a^ |
| University-entrance diploma^2^ | 0.34 | 0.35^a^ |
| Brandenburg | 0.03 | 0.03^b^ |
| Berlin | 0.04 | 0.04^b^ |
| Baden-Wuerttemberg | 0.14 | 0.13^b^ |
| Bavaria | 0.16 | 0.16^b^ |
| Bremen | 0.01 | 0.01^b^ |
| Hesse | 0.08 | 0.08^b^ |
| Hamburg | 0.02 | 0.02^b^ |
| Mecklenburg-West Pomerania | 0.02 | 0.02^b^ |
| Lower Saxony | 0.1 | 0.10^b^ |
| North Rhine-Westphalia | 0.21 | 0.22^b^ |
| Rhineland Palatinate | 0.05 | 0.05^b^ |
| Schleswig Holstein | 0.03 | 0.04^b^ |
| Saarland | 0.01 | 0.01^b^ |
| Saxony | 0.05 | 0.05^b^ |
| Saxony-Anhalt | 0.03 | 0.03^b^ |
| Thuringia | 0.03 | 0.03^b^ |
| Observations | 10,251 |  |

*Notes:* Smaragd Survey, Wave 3, January 2022. The mean values of the Casa Monitor variables are weighted by sampling weights. ^1^Values refer to 2021. ^2^Values refer to 2019. Sources of administrative data are
^a^Statistisches Bundesamt (Destatis), 2023 | Status: 2024/07/15, table codes 12211-0001, 12211-9012 , and 13111-0004.  ^b^Laufende Raumbeobachtung des BBSR - INKAR, Ausgabe 03/2024. Hrsg.: Bundesinstituts für Bau-, Stadt- und Raumforschung (BBSR), Bonn (<https://www.inkar.de/>).
^c^Verband der Ersatzkassen (vdek), 2024: Daten zum Gesundheitswesen: Versicherte, Status 2024/04/02, <https://www.vdek.com/presse/daten/b_versicherte.html>, last access on 2024/07/17.

**Table A2 Additional Variable info**

| **Variable** | | **Type** | **Information** |
| --- | --- | --- | --- |
| Days federal emergency brake* | | numeric | Days with active federal emergency brake at county level in the period 23 April 2021 to 30 June 2021. |
| Days with closed schools* | | numeric | Days of school closures due to federal emergency breaks at the county level in the period 23 April 2021 to 30 June 2021. |
| Male | | binary indicator | 1 if respondent indicates to be male and 0 otherwise. |
| Age | | numeric | Self-reported age of respondent |
| Fulltime | | binary indicator | 1 if respondents state to work at least 35 hour per week and 0 otherwise. |
| Education | | categorical | Self-reported highest achieved education level. |
|  | Basic track or less |  | Lowest educational attainment Hauptschule or no diploma |
|  | Intermediate |  | Educational diploma qualifying for most vocation training (*Realschule*) |
|  | University-entrance diploma |  | Highest school diploma |
| German | | binary indicator | Self-reported German citizenship |
| Private health insurance | | binary indicator | 1 if respondent reported to have private health insurance, 0 otherwise. |
| Married | | binary indicator | 1 if respondent reported to be married, 0 otherwise. |
| Party | | categorical | Self-reported preference for voting in a hypothetical federal election taking place next Sunday. |
|  | Christian democrats (CDU/CSU) | binary indicator | constructed from categorical party variable |
|  | Social democrats (SPD) | binary indicator | constructed from categorical party variable |
|  | Green party | binary indicator | constructed from categorical party variable |
|  | Liberal party (FDP) | binary indicator | constructed from categorical party variable |
|  | Left-wing (Die Linke) | binary indicator | constructed from categorical party variable |
|  | Right-wing (AfD) | binary indicator | constructed from categorical party variable |
|  | Other | binary indicator | constructed from categorical party variable |
|  | Nonvoter | binary indicator | constructed from categorical party variable |
| Working from home (WFH) possible | | binary indicator | 1 if the respondent's work could be theoretically done from home, 0 otherwise |
| WFH | | categorical | Those theoretically able to work from home were asked about their actual options for working from home |
|  | Not possible | binary indicator | constructed from categorical WFH |
|  | Without restrictions | binary indicator | constructed from categorical WFH |
|  | Only during WFH duty | binary indicator | constructed from categorical WFH |
|  | With restrictions | binary indicator | constructed from categorical WFH |
| High building | | binary indicator | 1, if respondent lives in a skyscraper |
| Migration share in city district | | numeric | Migration share based on inhabitants in the city district of the respondent |
| Unemployment rate city district | | numeric | unemployment rate of the dependent civil labour force in the city district of the respondent |
| Share high school degree | | numeric | Share high school degree in the city of the respondent |

*Notes:* Smaragd Survey, wave 3, January 2022. *Self collected.

**Table A3 Variance inflation factors**

|  | | **(1)** | **(2)** | **(3)** | **(4)** | **(5)** |
| --- | --- | --- | --- | --- | --- | --- |
| Vaccinated (self) | |  |  | 1.67 |  | 1.64 |
| Days federal emergency brake | | 1.76 | 1.88 | 1.88 | 1.69 | 1.69 |
| Days with closed schools | | 2.24 | 2.8 | 2.8 | 2.23 | 2.26 |
| Male | | 1.54 | 1.68 | 1.68 | 1.62 | 1.63 |
| Age | | 1.14 | 1.29 | 1.29 | 1.29 | 1.3 |
| Fulltime | | 1.49 | 1.78 | 1.79 | 1.53 | 1.53 |
| Educ. | Basic track or less |  |  |  |  |  |
|  | Intermediate | 1.89 | 2.26 | 2.26 | 2 | 2.02 |
|  | University-entrance diploma | 2.21 | 2.48 | 2.52 | 2.38 | 2.39 |
| German | | 1.27 | 1.36 | 1.38 | 1.34 | 1.34 |
| Private health insurance | | 1.15 | 1.25 | 1.27 | 1.2 | 1.24 |
| Married |  | 1.11 | 1.16 | 1.17 | 1.21 | 1.22 |
| Party | Christian democrats (CDU/CSU) |  |  |  |  |  |
|  | Social democrats (SPD) | 1.62 | 1.73 | 1.73 | 1.63 | 1.66 |
|  | Green party | 1.56 | 1.59 | 1.59 | 1.62 | 1.63 |
|  | Liberal party (FDP) | 1.41 | 1.45 | 1.47 | 1.59 | 1.59 |
|  | Left-wing (Die Linke) | 1.54 | 1.8 | 1.81 | 1.78 | 1.8 |
|  | Right-wing (AfD) | 1.8 | 2.01 | 2.27 | 2.12 | 2.2 |
|  | Other | 1.83 | 2.11 | 2.12 | 1.92 | 1.92 |
|  | Nonvoter | 1.58 | 1.42 | 1.43 | 1.84 | 1.89 |
| Working from home (WFH) possible | | 6.66 | 8.71 | 8.79 | 6.23 | 6.43 |
| WFH | WFH not possible |  |  |  |  |  |
|  | Without restrictions | 4.43 | 5.86 | 5.87 | 3.84 | 3.9 |
|  | Only during wfh duty | 3.13 | 4.15 | 4.15 | 3.03 | 3.06 |
|  | With restrictions | 2.53 | 2.9 | 2.9 | 2.44 | 2.45 |
| High building | | 1.29 | 1.38 | 1.4 | 1.35 | 1.35 |
| Migration share in city district | | 3.15 | 3.07 | 3.08 | 3.51 | 3.52 |
| Unemployment rate city district | | 2.51 | 3 | 3.15 | 2.59 | 2.63 |
| Share high school degree | | 1.78 | 1.89 | 1.91 | 1.92 | 1.93 |

*Notes:* Smaragd Survey, Wave 3, January 2022, Wave 3 January 2022. Variance inflation factors after regressions in Table 2. Federal state binary indicators are also included. Column (1) refers to the regression in column (1) of Table 2. and so on.

**Table A4 Baseline regressions without interactions**

|  | | **Own** | **Kids U12** | | **Kids 12-17** | |
| --- | --- | --- | --- | --- | --- | --- |
|  |  | **(1)** | **(2)** | **(3)** | **(4)** | **(5)** |
| Vaccinated (self) | |  |  | 0.590*** |  | 0.662*** |
|  | |  |  | (0.050) |  | (0.035) |
| Days federal emergency brake | | 0.014 | 0.020 | 0.012 | 0.031** | 0.016 |
|  | | (0.009) | (0.020) | (0.019) | (0.015) | (0.013) |
| Days with closed schools | | 0.035*** | -0.002 | -0.019 | 0.033* | 0.001 |
|  | | (0.010) | (0.023) | (0.021) | (0.017) | (0.014) |
| Male | | 0.038** | 0.038 | 0.048 | -0.061** | -0.106*** |
|  | | (0.017) | (0.038) | (0.035) | (0.030) | (0.026) |
| Age | | -0.025*** | 0.097*** | 0.096*** | 0.013 | 0.032*** |
|  | | (0.007) | (0.019) | (0.017) | (0.013) | (0.011) |
| Fulltime | | 0.010 | 0.057 | 0.013 | 0.061** | 0.062** |
|  | | (0.017) | (0.042) | (0.030) | (0.030) | (0.026) |
| Educ. | Basic track or less | reference | | | | |
|  | Intermediate | 0.031* | 0.177*** | 0.187*** | 0.034 | -0.02 |
|  |  | (0.019) | (0.047) | (0.043) | (0.034) | (0.030) |
|  | University-entrance diploma | 0.008 | 0.154*** | 0.207*** | 0.054 | 0.011 |
|  |  | (0.020) | (0.048) | (0.044) | (0.037) | (0.033) |
| German | | -0.065*** | -0.032 | 0.030 | 0.052 | 0.076** |
|  | | (0.021) | (0.047) | (0.044) | (0.040) | (0.034) |
| Private health insurance | | -0.088*** | -0.093** | -0.020 | -0.152*** | -0.054* |
|  | | (0.020) | (0.042) | (0.039) | (0.037) | (0.032) |
| Married | | 0.038** | -0.037 | -0.016 | 0.003 | -0.027 |
|  | | (0.015) | (0.036) | (0.033) | (0.028) | (0.024) |
| Party | Christian democrats (CDU/CSU) | reference | | | | |
|  | Social democrats (SPD) | 0.064*** | -0.059 | -0.040 | 0.105** | 0.014 |
|  |  | (0.024) | (0.054) | (0.049) | (0.043) | (0.038) |
|  | Green party | 0.01 | 0.119** | 0.150*** | 0.114** | 0.080* |
|  |  | (0.027) | (0.059) | (0.054) | (0.051) | (0.044) |
|  | Liberal party (FDP) | -0.056* | -0.196*** | -0.079 | -0.037 | 0.003 |
|  |  | (0.030) | (0.068) | (0.063) | (0.055) | (0.047) |
|  | Left-wing (Die Linke) | -0.146*** | -0.246*** | -0.134* | -0.039 | 0.077 |
|  |  | (0.037) | (0.078) | (0.072) | (0.067) | (0.058) |
|  | Right-wing (AfD) | -0.242*** | -0.662*** | -0.408*** | -0.156*** | -0.01 |
|  |  | (0.027) | (0.061) | (0.060) | (0.047) | (0.042) |
|  | Other | -0.02 | -0.277*** | -0.236*** | -0.015 | -0.037 |
|  |  | (0.022) | (0.052) | (0.048) | (0.040) | (0.035) |
|  | Nonvoter | -0.295*** | -0.337*** | -0.203** | -0.173*** | -0.021 |
|  |  | (0.032) | (0.094) | (0.087) | (0.056) | (0.049) |
| Working from home (WFH) possible | | 0.216*** | 0.057 | -0.065 | 0.102 | -0.082 |
|  | | (0.036) | (0.098) | (0.090) | (0.062) | (0.055) |
| WFH | not possible | reference | | | | |
|  | Without restrictions | -0.151*** | -0.071 | 0.050 | -0.089 | 0.047 |
|  |  | (0.039) | (0.103) | (0.095) | (0.068) | (0.059) |
|  | Only during wfh duty | -0.130*** | -0.006 | 0.078 | -0.128* | -0.006 |
|  |  | (0.041) | (0.109) | (0.101) | (0.072) | (0.062) |
|  | With restrictions | -0.128*** | 0.067 | 0.158 | 0.065 | 0.154** |
|  |  | (0.044) | (0.114) | (0.105) | (0.080) | (0.070) |
| High building | | 0.092*** | 0.058 | 0.032 | 0.06 | 0.069 |
|  | | (0.031) | (0.076) | (0.069) | (0.060) | (0.052) |
| Migration share in city district | | -0.013 | 0.046* | 0.016 | -0.115*** | -0.095*** |
|  | | (0.012) | (0.027) | (0.025) | (0.021) | (0.018) |
| Unemployment rate city district | | -0.056*** | -0.075*** | -0.023 | -0.031* | 0.002 |
|  | | (0.010) | (0.021) | (0.020) | (0.016) | (0.014) |
| Share high school degree | | -0.035*** | -0.009* | 0.010 | -0.026 | -0.004 |
|  | | (0.010) | (0.020) | (0.019) | (0.018) | (0.016) |
| Federal state binary indicators | | yes | Yes | Yes | yes | yes |
| Constant | | 0.974*** | 0.560*** | -0.158 | 0.413*** | -0.150 |
|  | | (0.046) | (0.116) | (0.123) | (0.105) | (0.095) |
| Observations | | 1817 | 762 | 762 | 1145 | 1145 |
| F | | 18.459 | 10.439 | 15.487 | 11.028 | 23.325 |

*Notes:* Smaragd Survey, Wave 3/January 2022. Weighted regressions. Standard errors are in parentheses; * (p<0.1), ** (p<0.05), *** (p<0.01); Continuous variables are standardized.

**Table A5 Multinomial logit, marginal effects**

|  | | Yes | | Not yet decided | | No | |
| --- | --- | --- | --- | --- | --- | --- | --- |
| Days federal emergency brake | | 0.047*** | (0.017) | -0.067*** | (0.016) | 0.020 | (0.015) |
| Days with closed schools | | 0.026 | (0.021) | -0.046** | (0.019) | 0.020 | (0.017) |
| Male | | 0.095*** | (0.033) | -0.118*** | (0.030) | 0.023 | (0.030) |
| Age | | 0.062*** | (0.017) | 0.014 | (0.016) | -0.076*** | (0.015) |
| Fulltime | | -0.056 | (0.036) | 0.136*** | (0.033) | -0.080*** | (0.031) |
| Educ. | Basic track or less | reference | | | | | |
|  | Intermediate | 0.065 | (0.041) | 0.033 | (0.039) | -0.098*** | (0.035) |
|  | University-entrance diploma | 0.156*** | (0.043) | -0.101*** | (0.039) | -0.055 | (0.038) |
| German |  | -0.001 | (0.042) | -0.070** | (0.035) | 0.072* | (0.039) |
| Private health insurance | | -0.026 | (0.038) | -0.028 | (0.035) | 0.055 | (0.034) |
| Married |  | -0.053 | (0.032) | 0.021 | (0.030) | 0.032 | (0.028) |
| Party | Christian democrats (CDU/CSU) | reference | | | | | |
|  | Social democrats (SPD) | 0.161*** | (0.051) | -0.256*** | (0.047) | 0.094** | (0.038) |
|  | Green party | 0.216*** | (0.055) | -0.165*** | (0.055) | -0.050* | (0.029) |
|  | Liberal party (FDP) | -0.061 | (0.063) | -0.107* | (0.063) | 0.168*** | (0.050) |
|  | Left-wing (Die Linke) | -0.021 | (0.076) | -0.243*** | (0.064) | 0.263*** | (0.068) |
|  | Right-wing (AfD) | -0.339*** | (0.048) | -0.327*** | (0.044) | 0.665*** | (0.044) |
|  | Other | -0.090* | (0.047) | -0.162*** | (0.045) | 0.252*** | (0.038) |
|  | Nonvoter | -0.170** | (0.086) | -0.015 | (0.091) | 0.185*** | (0.071) |
| Working from home (WFH) possible | | -0.101 | (0.084) | 0.219*** | (0.068) | -0.118 | (0.075) |
| High building |  | -0.085 | (0.063) | 0.166*** | (0.053) | -0.08 | (0.055) |
| Share high school degree | | 0.053*** | (0.019) | -0.067*** | (0.018) | 0.014 | (0.017) |
| Migration share in city district | | 0.024 | (0.025) | -0.014 | (0.024) | -0.010 | (0.022) |
| Unemployment rate city district | | -0.073*** | (0.022) | 0.047** | (0.021) | 0.026 | (0.017) |
| Observations | | 1073 | | 1073 | | 1073 | |

*Notes:* Smart survey, Wave 3, January 2022. Average marginal effects after weighted multinomial logit regressions. The outcome variable is willingness to vaccinate children aged between 5 and 11 years. The sample only includes parents who are vaccinated and have children aged between 5 and 11 years. Standard errors are in parentheses; * (p<0.1), ** (p<0.05), *** (p<0.01). Continuous variables are standardized.
